# Electrophysiological and Behavioral Markers of Hyperdopaminergia in DAT-KO Rats

**DOI:** 10.3390/biomedicines12092114

**Published:** 2024-09-17

**Authors:** Zoia Fesenko, Maria Ptukha, Marcelo M. da Silva, Raquel S. Marques de Carvalho, Vassiliy Tsytsarev, Raul R. Gainetdinov, Jean Faber, Anna B. Volnova

**Affiliations:** 1 Institute of Translational Biomedicine, Saint Petersburg State University, Saint Petersburg 199034, Russia; 2 Centre for Youth Mental Health, Faculty of Medicine, Dentistry and Health Sciences, The University of Melbourne, Victoria 3010, Australia; ptukhamaria@yandex.ru; 3 Department of Biophysics, Escola Paulista de Medicina, Universidade Federal de São Paulo, São Paulo 04039-032, Brazil; 4 Department of Anatomy and Neurobiology, University of Maryland School of Medicine, Baltimore, MD 21201, USA; 5 Saint Petersburg University Hospital, Saint Petersburg 190121, Russia; 6 Department of Neurology and Neurosurgery, Division of Neuroscience, Escola Paulista de Medicina, Universidade Federal de São Paulo, São Paulo 04023-900, Brazil; 7 Biological Faculty, Saint Petersburg State University, Saint Petersburg 199034, Russia

**Keywords:** behavior, dopamine, dopamine transporter (DAT), DAT-KO rats, local field potential (LFP), hyperdopaminergia

## Abstract

**Background/Objectives:** Dopamine dysfunction (DA) is a hallmark of many neurological disorders. In this case, the mechanism of changes in dopamine transmission on behavior remains unclear. This study is a look into the intricate link between disrupted DA signaling, neuronal activity patterns, and behavioral abnormalities in a hyperdopaminergic animal model. **Methods:** To study the relationship between altered DA levels, neuronal activity, and behavioral deficits, local field potentials (LFPs) were recorded during four different behaviors in dopamine transporter knockout rats (DAT-KO). At the same time, local field potentials were recorded in the striatum and prefrontal cortex. Correlates of LFP and accompanying behavioral patterns in genetically modified (DAT-KO) and control animals were studied. **Results:** DAT-KO rats exhibited desynchronization between LFPs of the striatum and prefrontal cortex, particularly during exploratory behavior. A suppressive effect of high dopamine levels on the striatum was also observed. Wild-type rats showed greater variability in LFP patterns across certain behaviors, while DAT-KO rats showed more uniform patterns. **Conclusions:** The decisive role of the synchrony of STR and PFC neurons in the organization of motor acts has been revealed. The greater variability of control animals in certain forms of behavior probably suggests greater adaptability. More uniform patterns in DAT-KO rats, indicating a loss of striatal flexibility when adapting to specific motor tasks. It is likely that hyperdopaminergy in the DAT-KO rat reduces the efficiency of information processing due to less synchronized activity during active behavior.

## 1. Introduction

One of the most abundant neurotransmitters, dopamine (DA), plays a key role in movement regulation, the reward system, and in many other neurological functions, including memory, motivation, mood, attention, and more. The dysfunction of dopaminergic systems is observed in many brain disorders, such as Parkinson’s disease (PD), attention deficit hyperactivity disorder (ADHD), schizophrenia, addictions (including alcohol and nicotine), and others [1,2,3,4,5,6]. 

In the neural system, DA is synthesized from tyrosine by the enzyme tyrosine hydroxylase; it is then accumulated in vesicles and, when necessary, released into the synaptic cleft. After interactions with receptors (D1-D5), DA must be removed from the cleft with reuptake by the dopamine transporter (DAT) [7,8,9,10,11]. This also serves to minimize the synthesis of new DA molecules, for which neurons have specific mechanisms to incorporate interstitial DA and reuse it [2,3]. Typically, it is the DA transporter that controls the balance of extracellular and intracellular DA concentration, preventing the onset of hyperdopaminergic or hypodopaminergic states.

Maintaining the balance of DA transmission is necessary to maintain the vital functions of the body. In the brain, there are four major dopaminergic pathways that play critical roles in the regulation of important physiological functions, such as movement, the control of prolactin synthesis, motivation, and others [12]. Dysregulation of the dopamine system occurs especially in the striatum (STR) and prefrontal cortex (PFC); these areas are the main focus of this study regarding motor control and cognitive deficits [13]. Dopaminergic imbalance is believed to contribute significantly to both positive and negative symptoms of schizophrenia. Hyperdopaminergic activity in subcortical regions, potentially due to increased release or transmission, is thought to underlie positive symptoms such as hallucinations and delusions [14]. Conversely, reduced D1 receptor activation in the PFC and caudate nucleus is hypothesized to contribute to negative symptoms such as anhedonia and amotivation [15]. This complex interplay has led to the “revised dopamine hypothesis” of schizophrenia, which proposes a coexistence of hypoactive DA transmission in the PFC and hyperactive transmission in the mesolimbic areas [14].

Therefore, a comprehensive study of DA physiology can pave the way for more accurate diagnoses and effective treatment strategies for psychiatric conditions. One promising approach involves the use of transgenic animals, specifically those with disrupted DAT genes, such as DAT-knockout (KO) mice and rats [1,16]. These animals, as demonstrated by [17,18], exhibit a functional state of hyperdopaminergia, manifested in pronounced spontaneous hyperactivity. 

The studies conducted by [16,19] revealed a characteristic profile within these DAT-KO rats: high extracellular DA levels in the STR coupled with a subsequent decrease in intracellular DA storage. Behaviorally, DAT-KO rats display several symptoms potentially linked to dopamine dysfunction, including hyperactivity, cognitive impairments, deficits in sensorimotor gating, and compulsive behaviors [20,21]. While not a perfect model for any single disease, DAT-KO rats offer a valuable tool as they encapsulate many prominent features of DA pathology [19].

The underlying electrophysiological neuronal activity also holds significant value for the understanding of the DA role. Specifically, the rhythmic fluctuations in neural local field potential (LFP) can be linked to distinct functional and behavioral states. Slow-wave oscillations, particularly in the delta frequency range (0.5–4 Hz), are associated with activity in subcortical areas critical for motivation, mood, and the reward system [12,22]. Conversely, faster brain rhythms, such as high beta (above 20 Hz) and gamma (above 40 Hz), reflect communication between different cortical regions (intercortical connections) and are amplified during behaviors demanding heightened attention [23,24]. Furthermore, theta waves (4–10 Hz) and synchronized activity between the PFC and hippocampus are known to increase during spatial navigation in rodents [22]. 

In the present study, we investigated patterns of neural activity (LFP) in an animal model with genetically manipulated levels of DA, considering possible mechanisms of hyperdopaminergia and its correlation with neuronal activity and behavior.

Our findings show how the elevated DA levels exhibited by DAT-KO rats disrupt the interplay between the STR, crucial for movement and reward, and the PFC, responsible for planning and execution. This desynchronization, particularly evident during exploration, suggests a potential mechanism behind the motor control issues observed in DAT-KO rats. Furthermore, our results reveal that in wild-type rats, brain activity patterns exhibit greater diversity across behaviors. However, in DAT-KO animals, these patterns become more homogenous, suggesting a loss of flexibility in the striatum’s ability to adapt to specific motor tasks.

## 2. Materials and Methods

### 2.1. Animals 

Behavior and local field potentials (LFP) were recorded for 14 adult male rats: DAT knockout (DAT-KO, *n* = 7) and wild-type (WT, *n* = 7) rats, males aged 3–4 months. Animal genotyping was performed according to the previously described protocol (Leo et al., 2018). All experimental procedures and protocols were approved by the Ethics Committee for Animal Research of Saint Petersburg State University, St. Petersburg, Russia, No. 131–03-10 of 22 November 2021. Rats were maintained in IVC cages (RAIR IsoSystem World Cage 500; Lab Products, Inc., Seaford, DE, USA) with free access to food and water, 50–70% relative humidity, and a 12 h light/dark cycle (light from 9 a.m.) at a temperature of 22 ± 1 °C. Experiments were carried out between 2 p.m. and 6 p.m.

### 2.2. Surgical Procedure

Electrode implantation procedures were performed using gas anesthesia (Biosthesia 300 system) using isoflurane. Local anesthesia with 0.5% Novocain was also used at the scalping site. Three electrodes were implanted using standard stereotaxic instruments (RWD Life Science Co., Shenzhen, Guangdong, China). Electrodes were placed in the following coordinates [25]: epidural reference electrode (7 mm posterior to bregma and 3 mm lateral to midline); intracerebral electrode to prefrontal cortex (PFC) (2 mm anterior to bregma, 1 mm lateral to midline, and 2.5 mm dorsoventral); striatal intracerebral electrode (0 mm anterior to bregma, 3 mm lateral to midline, and 5 mm dorsoventral). Intracerebral electrodes were used for LFP recordings (50 μm in diameter; 2.5 mm/5 mm in length; tungsten wire in perfluoroalkoxy polymer isolation), while an epidural screw was used for reference (1 mm in diameter; 1 mm in length; steel). Correct electrode placement was achieved by use of a micromanipulator in a stereotaxic frame, in which the animal’s head was fixed during the whole operating procedure. Electrodes were fixed on the skull with dental cement (Acrodent, Stoma, or Zhermapol, Poland).

### 2.3. Experimental Setup

After 3–5 days post surgery, the synchronous recording of behavior and electrical activity was performed in rats in free behavior. During the recording process, animals were placed in a 25 × 25 × 25 cm plexiglas box, which, along with the amplifier, was located in a Faraday cage. The experimental setting for electrophysiological recordings consisted of an amplifier (×1000 gain), a Power1401–3A (Cambridge Electronic Design, Cambridge, UK) data acquisition interface, and Spike 2 8.08 software (CED, Cambridge Electronic Design, Cambridge, UK); the sampling rate was 25,000 Hz. Behavior video recording was performed synchronously with LFP recording. Brain activity and behavior patterns were recorded twice on two different days for 40 min; types of behavior were identified from video recordings: exploring, rearing, grooming, and calm wakefulness. 

Parts of the LFP recordings corresponding to different types of behavior were analyzed in MATLAB^®®^. We identified the following patterns of behavior: “exploring”; “rearing”—the rat stands up on its hind legs to explore its environment; “grooming” with conservative sequencing patterns; and “wakefulness/calm”. During exploratory behavior, the rat explores the space of the experimental box without standing up on its hind legs, and during rearing—the rat stands up on its hind legs to explore its environment; self-grooming behavior—a rat cleaning its own body, rubbing their front paws on their face, then licking and rubbing the rest of their body down to their tail. Periods of rest and immobility during the experiment were also distinguished. Only parts of recordings where the animals were awake were used in subsequent analysis.

### 2.4. Signal Data Analysis

**Figure 1:**
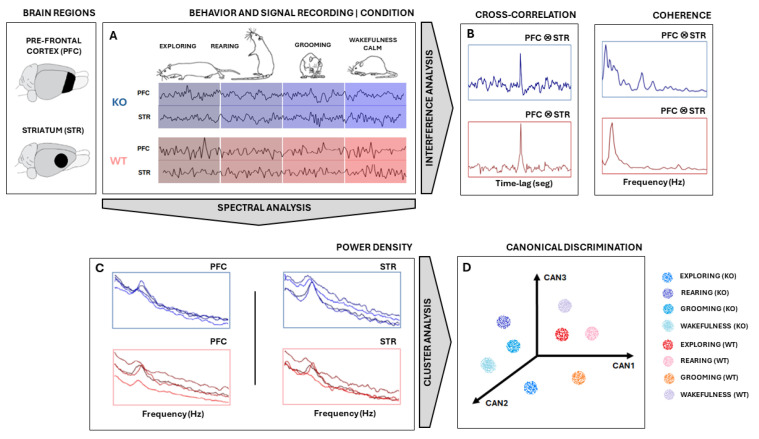
Experimental design. (**A**) Signal measured from STR and PFC electrodes in both KO and WT rats during specific behaviors: exploring, rearing, grooming, and wakefulness/calm. Signals were downsampled, segmented, filtered, and freed from artifacts. (**B**) The signal coherence and cross-correlation metric between PFC and STR activities were calculated. (**C**) Power Spectral Density (PSD) was computed using Welch’s technique and used as a feature input for Canonical Discriminant Analysis (CDA). (**D**) Each PSD can be thought of as a vector in a multidimensional space of frequencies. CDA performs a dimensional reduction conserving linear combinations (CAN1, CAN2, and CAN3) that maximize the correlation of intra-groups. Considering multiple epochs from each ‘behavior × animal type’, cloud clustering can be achieved. The same analysis was carried out with signals from the PFC and STR electrodes for both WT and KO groups. Illustrations of rats adapted from scidraw.io under a Creative Commons license.

All signal analyses and statistics were performed using MATLAB^®^ (version 9.2.0 R2018a, Mathworks Inc., Natick, MA, USA) software. Signals were measured in PFC and STR electrodes in the rat’s brains of two groups—KO and WT rats—during specific behaviors: exploring, rearing, grooming, and wakefulness/calm (Figure 1). First, the signal was segmented according to the type of electrode/type of rat/type of behavior combinations (Figure 1A). They were downsampled to 1000 Hz and bandpass filtered between 1 Hz and 50 Hz. Then, a search for and the removal of artifacts were carried out by visual inspection.

The power spectral density (PSD) of signal segments was performed with 5000 non-uniform (NFFT) points, and the PSDs means and standard deviations of the means were computed, according to PSD(w)=1/2π∑_(−∞)^∞R_xx (m)e^(−jwm); where w is the frequency range of interest, $R_{xx}\left(m\right)$ is the autocorrelation of a discretized $x\left[m\right],\;$*j* is the imaginary number [26]. All PSDs were shown in the range 0–30 Hz. To compare KO and WT PSDs, the confidence interval of their average was calculated with 95% certainty according to $IC=[\langle PSD\rangle \;\pm \;{1.96s}_{PSD}/\sqrt{n}]$, where $\langle PSD\rangle$ is the sampled PSD average, 1.96 is the coefficient of normality, $s_{PSD}$ is the sampled standard deviation, and *n* is the number of recording samples for each group [27]. PSDs were presented in graphics at the decibel scale relative to 1 mV2/Hz to enhance differences over power spectrum mean lines.

Following that, a multivariate analysis was carried out on PSD data (Figure 1C) to obtain the relationship between ‘behavior × electrode × animal type’ by using Canonical Discriminant Analysis (CDA). CDA on the PSDs allowed us to differentiate between the shapes of their power density distributions [28,29,30,31]. We used the one-way multivariate analysis of variance (MANOVA) function in MATLAB^®^(version 9.2.0 R2018a, Mathworks Inc., Natick, MA, USA): “manova1(X, group)”, where X is a trials-by-power/frequency matrix and “group” is a vector containing labels for each of the eight conditions (“exploring”, “rearing”, “grooming”, and “wakefulness/calm” for KO and WT). Manova performs a CDA to compute the differences between groups through F-statistics. 

CDA achieves dimensionality reduction by identifying orthogonal vectors (canonical variables) within the dependent space. The canonical variables capture the maximum possible variation between groups [32]. In other words, the first canonical variable, CAN1, represents the linear combination with the most significant separation between groups based on a one-way analysis of variance (ANOVA) F-statistic. CAN2 captures the maximum separation in the space orthogonal (uncorrelated) to CAN1, while CAN3 finds the maximum separation orthogonal to both CAN1 and CAN2, all based on F-statistic criteria.

To perform CDA, we grouped all PSDs calculated from each animal for both brain regions (STR and PFC) considering both conditions (DAT-KO and WT), as shown in Figure 1C,D. In this way, we were able to determine if these groups exhibited distinct, characteristic power shapes within specific frequency ranges. Hence, by considering each frequency range as a set of independent variables, if the PSD shapes hold enough information to differentiate each group defined by the categorical dependent variable, CDA should be able to identify and separate them (Figure 1D).

Further, we also calculate the signal coherence between PFC and STR activities (Figure 1B) according to $Coherence(w)=\frac{\;{\left|\langle {CPSD}_{PFC\;x\;STR}(w)\rangle \right|}^{2}}{\langle {\;PSD}_{PFC\;}(w)\rangle \langle {\;PSD}_{STR}(w)\rangle }$, where w is frequency, $\;\langle {\;PSD}_{PFC\;}(w)\rangle \;\;$ and $\langle {\;PSD}_{STR}(w)\rangle$ are the averaged power spectral densities of signal epochs from electrodes PFC and STR, respectively, and $\langle \;{CPSD}_{PFC\;x\;STR}(w)\rangle$ is the averaged cross power densities of simultaneous signal epochs from electrodes PFC and STR. To achieve coherent means and standard deviations, coherence was computed 50 times from the sampled epochs of each pool. Sampling was carried out randomly, without repetition, and in 50% of the epochs available.

Finally, we evaluated the similarity between recordings by applying the normalized cross-correlation metric $R_{xy}\left(\tau \right)=E\left[x\left(t\right)y\left(t+\tau \right)\right]/\sqrt{R_{xx}(0)R_{yy}(0)}$, where *t* is the time instant of signal epoch, *τ* is the time lag, and *E(.)* is the average. For each animal recording session, several smaller epochs of 2000 pts were taken. Since signs had different sizes, this procedure helped to homogenize all recordings and improved average stability across cross-correlation calculations. After this, the available epochs from PFC and STR were randomly sampled 50 times without replacement to calculate their cross-correlation, pair-to-pair. Then, the average and standard deviations across all pairwise cross-correlation outputs were taken. Finally, the means of the maximum values of cross-correlations (peaks) and their relative positions to zero (lags) of each KO and WT group were statistically compared using a *t*-test during all four specific behaviors of interest [33].

## 3. Results

**Figure 2:**
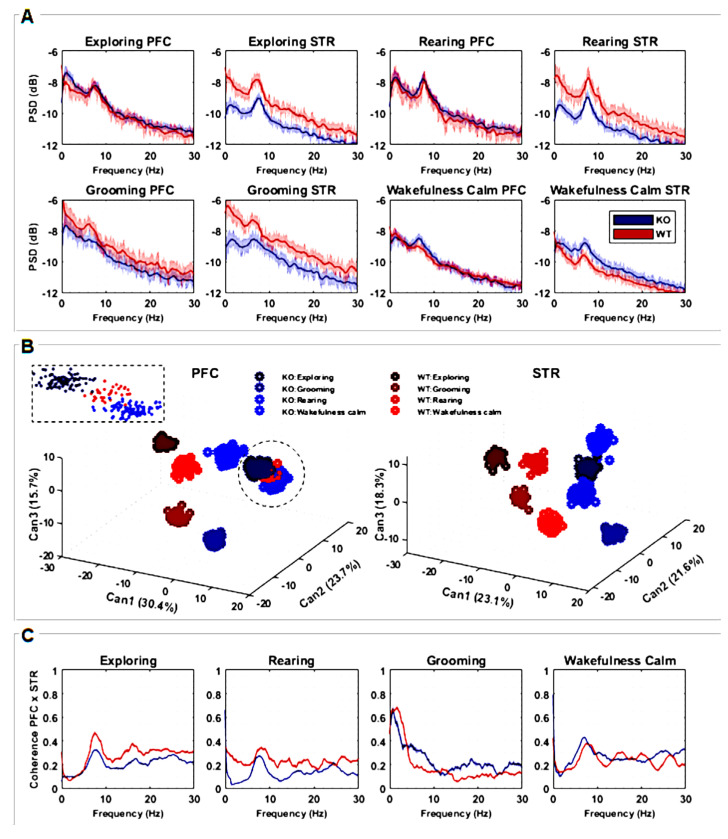
(**A**) Power spectral densities (PSDs) quantification considering recordings from PFC and STR brain regions of both groups, KO (*n* = 7) and WT (*n* = 7). The lines are the averaged PSDs with a 95% confidence interval in a frequency range between 0 and 30 Hz. PSDs are presented in decibels, scaled relative to 1 mV2/Hz to highlight the differences among power spectrum lines. (**B**) Discriminant canonical analysis (CDA) of PSDs, considering the four types of animal behavior for each separate brain region. (**C**) Coherence between recordings measured from PFC and STR brain regions in a frequency range between 0 and 30 Hz according to the four types of animal behavior. The plots represent the averaged coherence.

To evaluate the possible differences in electrophysiological features between DAT-KO and WT rats, we performed a series of analyses corresponding to LFP activities recorded from the dorsal striatum (STR) and prefrontal cortex (PFC). The characteristics of the signals recorded during four different behaviors (“exploring”, “rearing”, “grooming”, “wakefulness/calm”) were analyzed in the time-frequency domain (Figure 2). 

The power spectral density (PSD) of the striatal LFP signal was significantly lower in DAT-KO compared to WT during “exploring” (entire frequency range), “rearing”, and “grooming” behaviors (delta-alpha range). For the “wakefulness/calm” behavior, PSD was significantly greater for DAT-KO mainly in the theta and alpha ranges (Figure 2A). No significant differences between DAT-KO and WT were found in the PSD of the PFC signal for either of the four evaluated behaviors (Figure 2A) except for “grooming”, where PSD was slightly lower in the alpha range for the DAT-KO group in comparison to the WT group.

To evaluate how the shape of power density distributions is related to the intrinsic neurophysiological signaling processes underlying different behaviors, Canonical Discriminant Analysis (CDA) was implemented. The three most important combinations (CAN1, CAN2, and CAN3) were identified; the values (in %) denote how much each CAN was responsible for data clustering. Since CDA clusters emphasize the more correlated samples and distinguish between the most different ones, we were able to quantify how frequency modulates the signal distinctly for each situation. We used an F-test considering CAN cluster combinations for PFC, F(7;528) = 0.0313, *p* < 0.001, and STR, F(7;528) = 0.0308, *p* < 0.001. These results show that all clusters are significantly different from each other, as can be seen in Figure 2B.

For both brain regions (STR and PFC), it is possible to observe that different behaviors are separated in well-defined clusters. Particularly for the STR region, both groups (WT and KO) can be linearly separated, emphasizing how this area processes information differently from the PFC. For the PFC, except for the “rearing” behavior, WT and KO can also be linearly separated. It is worth mentioning that for this analysis, all rats were simultaneously considered, showing the consistency of the results. CDA clusters also enhance the results found with PSD analysis (Figure 2A,B), since corresponding statistical differences and similarities are amplified when considering all PSD variances and covariances together.

Coherence spectra with confidence intervals of 95% were also calculated (Figure 2C) in order to observe how the studied brain areas (STR and PFC) may be correlated in the frequency domain. Coherence peaks indicate interferences or even synchronization between STR and FPC signals along the frequency bands of interest. Since the coherence metric is normalized, values close to zero mean there is no coherence between STR and PFC regions, while values close to 1 mean the near total synchronization of neuronal activity. For “exploring” and “rearing” behaviors, peaks of coherence are observed in the theta and alpha ranges for both DAT-KO and WT, with coherence statistically greater for WT. During “grooming”, higher coherence can be seen for WT in delta and beta bands and lower theta and alpha bands. Finally, there are well-defined coherence peaks in the alpha band during “wakefulness/calm” behavior for DAT-KO and WT, with coherence greater for DAT-KO. Coherence is greater for WT only in the delta band, although peaks of coherence can be observed for frequencies of approximately 15 Hz, 20 Hz, and 27 Hz (Figure 2C).

**Figure 3:**
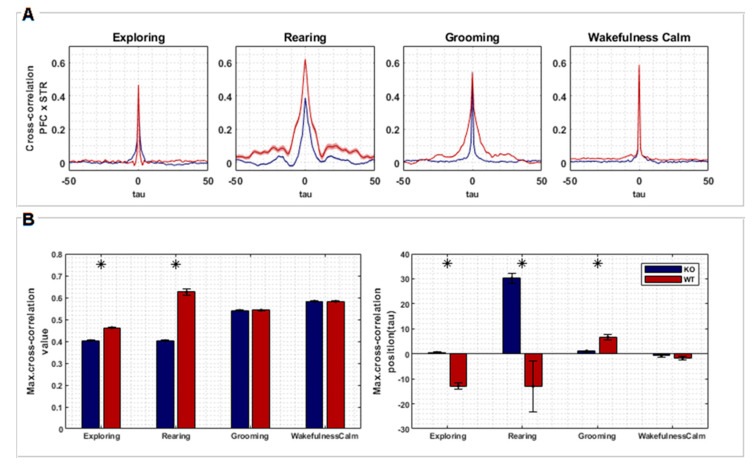
(**A**) Cross-correlation between signals recorded from PFC and STR regions of both groups, KO (*n* = 7) and WT (*n* = 7), according to each behavior (exploring, rearing, grooming, and wakefulness/calm). The plots represent the averaged cross-correlations of multiple epochs of the associated signals. Time lags along the X axis represent relative negative and positive displacements made between pairs of signals recorded from PFC and STR (each lag corresponds to 1 ms). (**B**) Statistical tests (*t*-test, two sample means) were performed comparing the maximum cross-correlation values of each group (left) and its respective lags (right). Asterisks represent a significant difference.

Cross-correlation analysis quantified the temporal similarity between recordings from two brain regions, PFC and STR. Figure 3A shows the average of all cross-correlation segments picked up corresponding to the four behaviors of interest. Although the differences in Figure 3A are not readily apparent, we found significant differences between KO and WT rats when comparing maximum correlation values and time delays between both groups (Figure 3B). 

The correlation between PFC and STR was higher for the WT group in comparison to the KO group during “exploring” (T= −20.033, *p* < 0.001) and “rearing” (T= −80.641, *p* < 0.001). However, no difference was found during “grooming” and “wakefulness/calm” (Figure 3B). Signals from PFC were found to be temporally lagged in relation to STR signals for WT rats during “exploring” (T= 10.007, *p* < 0.001) and “rearing” (T= 16.471, *p* < 0.001), but advanced during “grooming” behavior (T= −5.652, *p* < 0.001). On the other hand, signals from PFC were temporally advanced in relation to STR for KO rats during “rearing”. Finally, no temporal differences were found during the “wakefulness/calm” behavior (Figure 3B).

The WT group showed a higher temporal lag, comparing PFC and STR activity, during “exploring” and “rearing”. This effect can reflect a specific order of information processing, where the STR initiates a response that is then integrated by the PFC for a higher-level control. In DAT-KO rats, the temporal relationship seems less defined, with advanced PFC activity during “rearing”. This disruption in the timing of communication could contribute to the stereotypical behavior associated with hyperdopaminergia. No significant lag differences were observed during “grooming” and “wakefulness/calm”, suggesting these behaviors might rely less on coordinated PFC-STR activity.

Therefore, considering these results together, we can infer that hyperdopaminergia in DAT-KO rats can reduce information processing efficiency due to less synchronized activity during active behaviors. The altered order of information flow between STR and PFC can potentially interfere in decision-making and motor control.

## 4. Discussion

This study investigated the connection between the electrophysiological activity of STR and PFC and four different types of behavior, “exploring”, “rearing”, “grooming”, and “wakefulness/calmness”, in wild-type (WT) rats and dopamine transporter knockout (DAT-KO) rats. The results showed that hyperdopaminergia affects how STR and PFC communicate and affect or are affected by specific behaviors. In summary, DAT-KO rats exhibited (i) lower signal power in the striatum region, (ii) lower coherence between the striatum and prefrontal cortex, mainly during exploring and rearing behaviors, and (iii) shifted synchronization between the striatum and prefrontal cortex during grooming. 

Although it seems clear that DA imbalance impacts neuronal activity, no clear electrophysiological markers of dopaminergic transmission disorder states have been identified so far. Genetically modified animal models, such as DAT-KO, present a unique opportunity to study clinical correlates of specific physiological changes. Genetic deletion of the DAT gene in DAT-KO animals leads to pronounced hyperdopaminergia in the STR but not in PFC [16,19,34]. DAT-KO rats exhibit significant changes in learning and spatial memory, as well as pronounced motor hyperactivity, illustrating that DA imbalance between PFC and STR is critical for these functions [16,21,35,36]. 

In our previous study, DAT-KO rats showed significant decreases in power in the theta band in the prefrontal cortex, motor cortex, and striatum, with overall increases in power throughout the rest of the range [37]. Furthermore, we found this model to be characterized by generally lower coherence between STR and PFC when compared to WT [37]. However, these studies did not take into account the behavior of the animals during the experiment, focusing only on the state of wakefulness as a whole. 

Like all models, the DAT-KO model we used has not only advantages but also certain limitations. One of the key elements of dopamine (DA) neurotransmission is the DA transporter (DAT), a protein embedded in the cell membrane that is responsible for the reuptake of the transmitter. Changes in DAT function in DAT-KO rats are a key mechanism in various pathological conditions associated with hyperdopaminergy. Genetically modified DAT-deficient rats are characterized by elevated DA levels primarily in the striatum but not in PFC. It results in motor hyperactivity, hyperactivity, cognitive deficits, and other behavioral abnormalities. This provides a rationale for using DAT-KO rats as a convenient model for studying dopamine abnormalities, but the clinical picture in humans is more complex, and hyperdopaminergic states are not limited to elevated dopamine levels in the striatum.

In this work, we assessed the power spectrum and its synchronicity (with cross-correlation and coherence) of neuronal activity recorded from the cortex and striatum of DAT-KO and WT rats, taking into account four different behaviors (exploring, rearing, grooming, and wakefulness/calm). DAT-KO presented significantly lower values of signal power recorded from STR compared to WT rats across the whole frequency spectrum. This is true for all types of behavioral activity, excluding “wakefulness/calm” behavior. No such differences were observed for the signal obtained from the PFC. 

Specific behaviors can induce an increase in LFP power due to a DA release in a mutual causality process. However, as DAT-KO animals already have high levels of DA in their synaptic clefts, an increase in LFP power may not occur, even during the associated behaviors. This interpretation may also explain why the “wakefulness/calm” behavior was not characterized by differences in LFP power between the KO and WT groups, since this behavior does not demand enhanced motor activity. 

By applying Canonical Discriminant Analysis (CDA) to evaluate power spectral density in four behaviors across two groups, we demonstrated that all striatal PSD clusters related to KO behaviors are linearly distinguished from all PSD clusters related to WT behaviors. This could happen due to the different levels of DA in STR for each group, which affect the way brain oscillations are produced. Another interesting aspect is that in WT rats, PSD patterns are more dispersed than in KO rats, which is true across all behaviors. This suggests that an elevated level of DA somehow underlies greater homogeneity in neuronal activity of the striatum. 

In order to evaluate the interplay between PFC and STR in the frequency and time domains, we evaluated coherence (Figure 2C) and cross-correlation (Figure 3), respectively. Coherence metrics showed statistical differences between WT and KO, mainly for “exploring” and “rearing” behaviors (Figure 2C). During these behaviors, coherence between STR and PFC was lower for DAT-KO compared to WT. The coherence between STR and PFC during “grooming” and “wakefulness/calm” also differed between KO and WT. These observations are consistent with previous findings [37,38]. These results indicate that the link between PFC and STR, and therefore its role in the organization of active behavior, is disrupted in DAT-KO. Additionally, maximum cross-correlation values were found to be significantly higher in KO animals for “exploring” and “rearing”; however, no differences were found for “grooming” and “wakefulness/calm”. These results corroborate the findings of coherence, emphasizing that hyperdopaminergia disrupts the synchronization of STR and PFC during active motor behaviors. 

In summary, brain activity patterns in WT rats showed greater variance compared to DAT-KO rats. It suggests a kind of dampening effect of high DA in the STR, making activity patterns across different behaviors more similar. We also found a disruption in communication between the STR and the PFC in DAT-KO animals. Normally, these regions work in synchronicity to organize movements. However, hyperdopaminergia seems to decouple their activity, mainly during exploratory behaviors. This desynchronization could be a key factor underlying the motor control issues observed in DAT-KO rats. These findings are similar to what is seen in human diseases affecting the dopaminergic system.

Altered communication pathways within the brain demonstrated with EEG have been shown in human disorders associated with DA imbalance, such as Parkinson’s disease and ADHD [39]. For instance, studies on PD patients have shown a decrease in brainwave activity related to the beta band (alertness and focus) in the frontal and central regions of the brain and in the gamma bands (higher cognitive functions) across the central, parietal, and temporal brain regions [40]. This analysis also revealed differences between early and advanced stages of PD, where patients with advanced PD showed a further reduction in beta waves in the posterior central region, along with an increase in theta and delta-2 waves (slower brain waves) in the left parietal region [40]. These results highlight the direct link between changes in brainwave activity and the severity of dopaminergic dysfunction diseases. 

In DAT-KO mice [41], chronic hyperdopaminergia manifested in the form of hyperlocomotor behavior is accompanied by pronounced changes in LFP oscillations in the cortex and striatum. Data suggest that DA-related behavioral disorders may be mediated by changes in the coordinated activity of corticostriatal circuits. It seems that DA plays an important role in the precise modulation of synchrony generation mechanisms, which is corroborated by the results of the current study. Additionally, persistent hyperdopaminergia in DAT-KO mice has been shown to correlate with changes in the hippocampal theta oscillations during baseline behavioral periods [42] and to alter sleep-wake states [43].

The coherence of signals recorded from different parts of the brain is an important parameter that helps to identify functional synchronization (or lack thereof) between corresponding areas. Increased coherence in the theta band and reduced frontal gamma interhemispheric coherence have been reported in ADHD patients [44,45]. Adults with ADHD exhibit cortical hyperactivation manifested in decreased resting alpha power [46]. Higher delta power in the fronto-central region and higher theta/beta power in the bilateral frontotemporal region were also shown in ADHD patients [47]. Moreover, EEG studies showed that methylphenidate (pharmacotherapy for ADHD) increased alpha and beta activity in frontal regions and decreased delta and theta activity in occipital and parieto-occipital brain regions.

Many researchers have argued that schizophrenia may be associated with subcortical hyperdopaminergia, mainly in the STR. Although it is not the only cause of schizophrenia, it may underlie the exacerbation of positive symptoms. Electrophysiological activity in the PFC of patients with schizophrenia is characterized by higher levels of cortical response variability, and increased levels of DA may correlate with decreased PFC activity [48]. 

With the current study, we provide novel evidence on how dopamine affects brain communication and synchronization between cortical and subcortical structures. The discovery of differences in electrophysiological activity between WT and DAT-KO groups during specific behaviors paves the way for future investigations into the role of dopamine in neuropsychiatric disorders. The striking parallels between these findings and those observed in human dopamine-related disorders highlight the potential of this model. It can be a way for the development of novel therapeutic strategies for different conditions involving dopamine dysregulation, such as ADHD.

## Data Availability

All data presented during this study are included in this published article. The raw data used in this study are available on request from the corresponding author.
